# Towards a Better Performance in Facial Expression Recognition: A Data-Centric Approach

**DOI:** 10.1155/2023/1394882

**Published:** 2023-11-03

**Authors:** Christian Mejia-Escobar, Miguel Cazorla, Ester Martinez-Martin

**Affiliations:** ^1^Central University of Ecuador, P.O. Box 17-03-100, Quito, Ecuador; ^2^Institute for Computer Research, University of Alicante, P.O. Box 99. 03080, Alicante, Spain

## Abstract

Facial expression is the best evidence of our emotions. Its automatic detection and recognition are key for robotics, medicine, healthcare, education, psychology, sociology, marketing, security, entertainment, and many other areas. Experiments in the lab environments achieve high performance. However, in real-world scenarios, it is challenging. Deep learning techniques based on convolutional neural networks (CNNs) have shown great potential. Most of the research is exclusively model-centric, searching for better algorithms to improve recognition. However, progress is insufficient. Despite being the main resource for automatic learning, few works focus on improving the quality of datasets. We propose a novel data-centric method to tackle misclassification, a problem commonly encountered in facial image datasets. The strategy is to progressively refine the dataset by successive training of a CNN model that is fixed. Each training uses the facial images corresponding to the correct predictions of the previous training, allowing the model to capture more distinctive features of each class of facial expression. After the last training, the model performs automatic reclassification of the whole dataset. Unlike other similar work, our method avoids modifying, deleting, or augmenting facial images. Experimental results on three representative datasets proved the effectiveness of the proposed method, improving the validation accuracy by 20.45%, 14.47%, and 39.66%, for FER2013, NHFI, and AffectNet, respectively. The recognition rates on the reclassified versions of these datasets are 86.71%, 70.44%, and 89.17% and become state-of-the-art performance.

## 1. Introduction

Our facial gestures speak more than a thousand words. Among the dynamic activities of the human body, the muscular movements of the face have meaning and potential interpretation. Facial expressions associated with the emotional state of a person are considered universal and the main signal to manifest and infer our feelings and sensations [1, 2]. An important study [3] quantified the degree of influence of the elements involved in the communication of emotions, determining the nonverbal part (facial and body gestures) as the most influential with 55%, whereas the tone of voice with 38%, and only 7% for verbal language. In a conversational context, the exclusively verbal manifestation of anger or happiness must be accompanied by a facial gesture to convey the credibility and conviction of the interlocutor. Even the gesture would be enough to describe the emotion we are experiencing, as we often pay more attention to the face than to the words. The recent pandemic has shown that when a facial mask is present, the human capacity to infer emotions is reduced [4]. Therefore, facial expressions that communicate emotions are essential in daily life at the individual, interpersonal and social levels [5]. Apart from interacting with other people, we are increasingly surrounded by machines trying to imitate human behavior, so there is a need to interact. In near future, this will be a common practice and it is intended to make such interaction as natural as possible. In the same way that people can infer the emotional state of others from facial expressions, computers and robots may also be able to recognize expressions and interpret human emotions. In recent years, automatic facial expression recognition (FER) has become an important area of research and development to improve human-machine interaction (HMI), leading communication to a more emotional, affective, and intelligent level [6, 7]. This can be applied to many activities and fields such as human behavior, healthcare, medicine, psychology, psychiatry, marketing, digital advertisement, customer feedback assessment, video games, video security, video surveillance, mobile phone unlocking, crime investigation (lie detection), online learning, and automobile safety [8–11].

### 1.1. Problem

Humans can easily recognize facial expressions, however, it is still a challenge for machines [12]. Automatic FER is one of the key tasks in the field of computer vision. This problem has motivated competitions such as the one organized on the Kaggle platform [13]. A popular approach is to classify the facial expression in a static image of a human face and associate it with one of the seven basic universal human emotions: happiness, surprise, anger, sadness, fear, disgust, and neutral [14, 15]. Some models measure emotions with continuous values (e.g., valence and arousal). However, there are very limited annotated facial databases [16]. In contrast, for a discrete (categorical) model, a wider range of available datasets can be found. Deep learning is preferred for this task avoiding the high cost of time and effort of manually defining multiple and complex features of facial expressions. In particular, convolutional neural networks (CNNs) have shown promising results from different facial image datasets. Images captured in a specific and controlled environment (in the lab) are taken of a few people, do not present variations in environmental conditions, and gestures have a high degree of expressivity, so a good level of accuracy can be achieved. Another way is collecting images in real-world situations from the Internet, which is referred to as in the wild [17]. The heterogeneity of human faces, people less expressive than others, subtle differences between expressions, variations in head pose, different body postures, lighting changes in the environment, and occlusions, are some of the factors that make FER outside the laboratory a difficult task even for humans [9, 18–20].

### 1.2. Motivation

A deep learning solution consists of a model and data. The vast majority of work follows a model-centric approach, whose purpose is finding new algorithms to achieve better performance on a certain facial image dataset. Several CNN architectures have been proposed, both customized (created from scratch) and pretrained using transfer learning and fine tuning techniques. Each one tests different hyperparameters and includes regularization mechanisms such as data augmentation, dropout, and batch normalization [9]. In practice, this process is very time-consuming and has not achieved the aim of ideal performance. On the other side, there is research data-centric guided by the principle that data is the most important resource and its quality directly influences the performance of learning models. Very few studies have focused on improving FER datasets even though the same creators admit the problems in the quality of the data [8]. The lack of remarkable results of the model-centric approach, the little work focused on the data, and the premise that the data would be more important than the model, motivate us to propose a novel data-centric method to improve existing FER datasets to achieve better performance of recognition models.

### 1.3. Hypothesis

The quality of the dataset is a prerequisite for improving the accuracy of FER models. If the inherent drawbacks of the dataset are not reduced, it is very difficult to improve the performance of a FER system. In other words, better performance and higher accuracy are expected if the dataset is improved.

### 1.4. Method

Improving the main resource of a FER model, i.e., the dataset, implies improving the accuracy of the recognition. To validate our proposal, we used some representative datasets of this domain, which suffer from well-known problems such as imbalance, irrelevant images, and misclassified images. Our interest is to deal with misclassification, since balancing or removing irrelevant images would modify the size of the dataset. In contrast, a reclassification would generate a new distribution of the available images in a better-quality dataset. The strategy is a progressive refinement of the dataset over several trainings of the same CNN-based model. After each training, the prediction of all facial images is performed, and only the correct ones are selected to form the dataset for the next training. This process is repeated until there are few incorrect predictions, usually single-digit numbers. As a result, the last trained model achieves very high accuracy, so it is in charge of relabeling all the images of the original dataset. Therefore, a new distribution of the dataset is generated without altering its size or modifying the images. In the final step, the same CNN model is trained on the reclassified version of the dataset, and the accuracy is higher compared to the original dataset. The experiments performed in the present work show an increase of 20.45%, 14.47%, and 39.66% for the FER2013, NHFI, and AffectNet datasets, respectively. State-of-the-art performance was also achieved for these datasets.

### 1.5. Contributions

Our research work provides: (1) a novel data-centric method to reclassify the images of a dataset that allows a higher precision of a FER model, (2) a methodology applicable to other datasets from different domains and supported by computer tools, especially Python and deep learning libraries, and (3) a reclassified version of each dataset, which may be useful for further research, publicly available for FER2013 and NHFI, whereas for AffectNet this is not possible due to licensing restrictions.

The content of this work is organized as follows: Section 2 reviews the data-centric works.  Section 3 presents the FER datasets.  Section 4 describes in detail the methodology.  Section 5 explains the experimentation, and the results obtained in Section 6. Finally, Section 7 includes the conclusions and mentions future work.

## 2. Related Work

Our bibliographic search on improving the performance of FER in the wild using deep learning reports supremacy of model-centric research. This approach focuses on better architectures, hyperparameter tuning, and regularization techniques [21]. However, no significant progress can be expected when the data used are not reliable. On the other hand, data-centric efforts are scarce. There are few studies that deal with the dataset to improve the performance of a FER system. After analyzing the related literature, we can say the techniques frequently used under this approach include: image preprocessing, removing noise, deleting images with errors, data augmentation, and reclassification. For instance, Liu et al. [11] analyzed expression recognition considering the importance of data preprocessing by improving the image contrast. More discriminative facial features are obtained using a hybrid method for extraction, and a classification network combining EGG-16 and ResNet. Experiments on three benchmark datasets: CK+, FER2013, and AR achieved state-of-the-art recognition rates: 98.6%, 94.5%, and 97.2%, respectively. Kim et al. [22] designed an image and video preprocessing system called FIT (facial image threshing) machine capable of eliminating irrelevant facial images, cropping, resizing, and reorganizing the classification of facial images before training the Xception algorithm, improving the validation accuracy by 16.95% with the FER2013 dataset. Mazen et al. [8] applied the following operations on the dataset: (1) nonface images, text images, and profile images are deleted, (2) wrongly labeled images are relabeled using a CNN, and (3) data augmentation to overcome the class imbalance, generating new face images for the minority classes with a cycle generative adversarial network (CycleGAN). As a result, the average test accuracy was increased from 64% for the original FER2013 dataset to 91.76% for the modified balanced version. The cited works address the preprocessing of the dataset before the training of a model, however, the operations applied to change the total number of images either by removing or augmenting. In addition, the images are modified by cropping, resizing, or retouching the contrast. Our goal is to preserve the images and size of the dataset, so we focus on misclassification, one of the most influential problems in the lower performance of the FER models. For instance, Kim and Wallraven [23] presented a study of the quality of the labeling on AffectNet. Due to the large size of the dataset, a subset with a total of 800 difficult-to-recognize images of the different categorical expressions was selected to be relabeled by 13 human annotators. After the crowd reannotation, 83.25% of the total number of votes did not match the original dataset labels. In addition, the predictions of several ResNets trained on the original AffectNet are compared with the labels assigned by the human crowd, finding that there is no good coincidence for categorical expression. This pilot test suggests the low labeling quality of the original dataset for these difficult facial images, influencing the poor performance of a deep learning model. It is mentioned that more extensive reannotation work is in progress to check more accurate performance, however, manual annotation demands great effort and time. Our work does not require any kind of preparation or modification of the images, and avoids decreasing or increasing their number. It aims to automatically reclassify images to reduce intraclass variability and interclass overlapping of the original dataset. As a consequence, improve recognition performance.

## 3. Datasets

There are multiple image datasets created for automatic emotion recognition based on facial expressions. We have considered FER2013, AffectNet, and NHFI (natural human face image), mainly due to availability, size, image format, and categories of facial expressions.

### 3.1. Characteristics

The FER2013 dataset (created by Pierre-Luc Carrier and Aaron Courville) and AffectNet (Ali Mollahosseini, Behzad Hasani, and Mohammad H. Mahoor) are standards taken as benchmarks for competitions [24], whereas NHFI (Sudarshan Vaidya) is a novel dataset, created for the purpose of providing more data with better manual annotation, which we propose to analyze in the present study.  Table 1 summarizes the most relevant characteristics of these datasets.

The quality of the datasets is more affected as the size of the dataset increases, so we selected a dataset at different scales: small (thousands of images), mid (tens of thousands), and large scale (hundreds of thousands). The facial images included are static, not video sequences, with a 2D or flat appearance, in contrast to the 3D images that generate a perception of depth [1]. Each image has a facial expression category assigned to it, this is a task performed entirely by humans, except for AffectNet, where one part was manually annotated and the rest automatically annotated using a neural network trained on all manually annotated training set samples [16]. The datasets are not balanced, i.e., they do not have the same number of images for each category, or at least a similar number. This drawback is discussed later. To examine the influence of image color and size on recognition performance, we have images in grayscale and RGB mode, as well as in small and medium sizes. JPG and PNG are standard image formats and are easy to convert to each other. The datasets encompass difficult naturalistic conditions (in the wild), with images far from a controlled environment, closer to reality, different lighting levels, ages, poses, intensity of expression, and occlusions, making recognition a challenging task [19].

### 3.2. Acquisition

The FER2013 (https://www.kaggle.com/datasets/deadskull7/fer2013) and NHFI (https://www.kaggle.com/datasets/sudarshanvaidya/random-images-for-face-emotion-recognition) datasets are publicly available in Kaggle, whereas AffectNet requires permission for use via a request form to the authors (request form: mohammadmahoor.com/affectnet-request-form/). FER2013 can be obtained in a comma-separable value (CSV) format whose columns represent the following attributes: a value between 0 and 6 for each of the 7 possible emotions (0: angry, 1: disgust, 2: fear, 3: happy, 4: neutral, 5: sad, and 6: surprise), a list of 2304 integer values, each equivalent to one pixel of the image of size 48 × 48, and finally the subset to which it belongs: training or test. Since the images are not directly visible, we used a Python script with the Pandas and NumPy libraries to read the file, store the integer values as pixel arrays, and convert them to image files. A total of 35886 images are obtained after transforming the pixel arrays to image files in JPG format, in grayscale and with a resolution of 48 × 48 pixels, divided into two subsets: training and test, 28708 and 7178 images, respectively. Each subset includes 7 folders, each one for a particular type of facial expression. NHFI downloading is a compressed file, which after decompression generates 8 folders, whose names are practically the same as the previous dataset, only the “contempt” category is excluded for a fair comparison. Inside each folder are images in PNG format. In the case of AffectNet, the link provided in response to the request allows for the download of two compressed archives for training and validation. After the extraction of each archive, an “images” folder containing the JPG files and another one called “annotations” containing the NPY files of the corresponding labels are created. We developed a Python script (github.com/cimejia/FER-datasets/blob/main/createAffecnet.py) to read the facial expression category from the NPY file and move the JPG file to the corresponding folder. It is worth mentioning that AffectNet has two versions of the dataset, we used the small one containing only the manually annotated images with 8 labels (but contempt is omitted) released in March 2021. The full AffectNet dataset is huge (122 GB) and a specific request is necessary [16].

### 3.3. Drawbacks

Automatic collection from the Internet and label crowdsourcing are the main reasons for the quantity and quality drawbacks of FER datasets. Regarding quantity, the major disadvantage is the imbalance, even with categories that largely exceed the number of facial images in other categories. On the other hand, the quality of the content is highly affected by the presence of irrelevant images and misclassification. These problems are widely mentioned in the literature and increase as the size of the dataset grows [8].

#### 3.3.1. Imbalance

An imbalanced dataset could lead to a recognition model biased in favor of the majority classes. Having the same number of images per category is a difficult task. Facial images are usually sourced from the Internet and collected manually or automatically through browser plug-ins or programming scripts. These images are posted by people who tend to show smiling or happy faces, so this category predominates, in contrast to categories such as disgust, anger, or sadness, which users do not usually post.  Table 2 indicates the number of images per facial expression category in each dataset.

**Figure 1:**
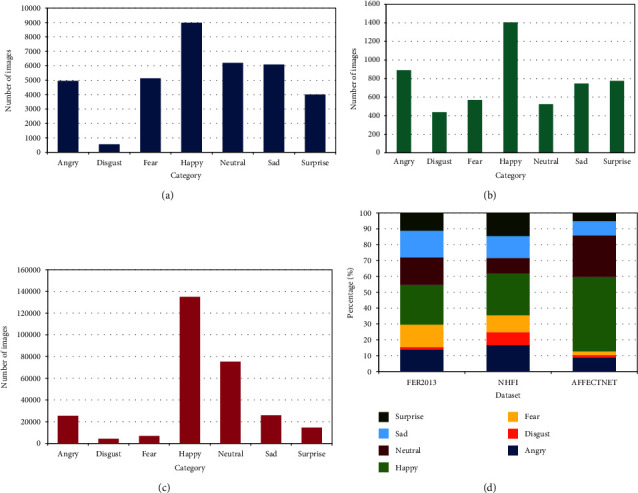
Imbalance in (a) FER2013; (b) NHFI; and (c) AffectNet; (d) overall.

All three datasets show a significant imbalance (Figure 1). In FER2013 (Figure 1(a)), the “happy” category predominates, and the “disgust” category has few samples, and it is approximately regular for the rest of the categories. NHFI (Figure 1(b)) presents a similar behavior, but is less irregular. In AffectNet (Figure 1(b)), the difference in the number of images between all categories is much more pronounced.

Comparing the distributions on the same scale (Figure 1(d)), the imbalance is much more significant in AffectNet. A common pattern is the higher number of samples for the happy category and the lowest number for the disgust category. As mentioned before, this is because people tend to post images of happy faces and avoid showing other types of expression.

#### 3.3.2. Misclassification and Irrelevant Images

Here, we join both problems related to the content of the datasets. Misclassification or mislabeling refers to placing facial images in the wrong directories. Among the factors that lead to this problem are: (a) emotions are subjective, it is common that two people to have different opinions on the same facial image, (b) there are slight differences between certain facial expressions, e.g., fear and surprise, disgust and anger, and contempt and sadness, (c) the degree of expressiveness varies from person to person, so gestures may appear exaggerated in one case and inhibited in others, and (d) human beings can feel multiple emotions in a given instant, something that is difficult to combine in a facial expression and can be confusing, e.g., smiling carrying tears is a combined emotion mistaken for sadness [9, 25, 26]. As irrelevant images are those with watermarks, occlusions, no faces, poorly visible or very dark, cartoons, text or symbols, half-side, sleeping faces or closed eyes, cropped, rotated, retouched, and duplicated images. It is important to check for these drawbacks in each dataset, however, an exhaustive manual and visual review of a large number of images are impractical. We designed the following procedure to easily locate such errors.

**Figure 2:**
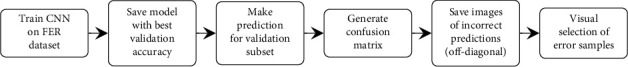
Workflow for selecting and showing some error samples.

**Figure 3:**
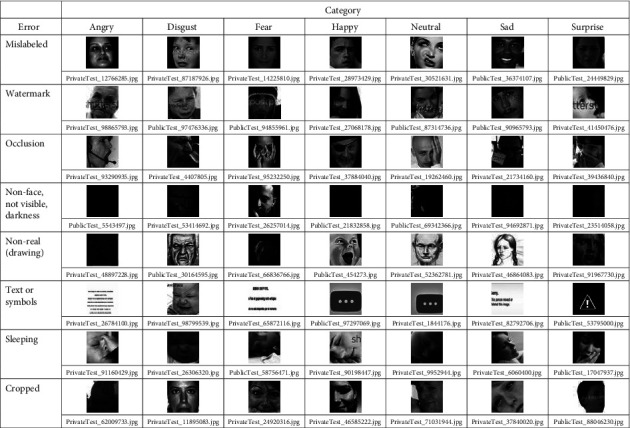
Some errors in the FER2013 dataset.

**Figure 4:**
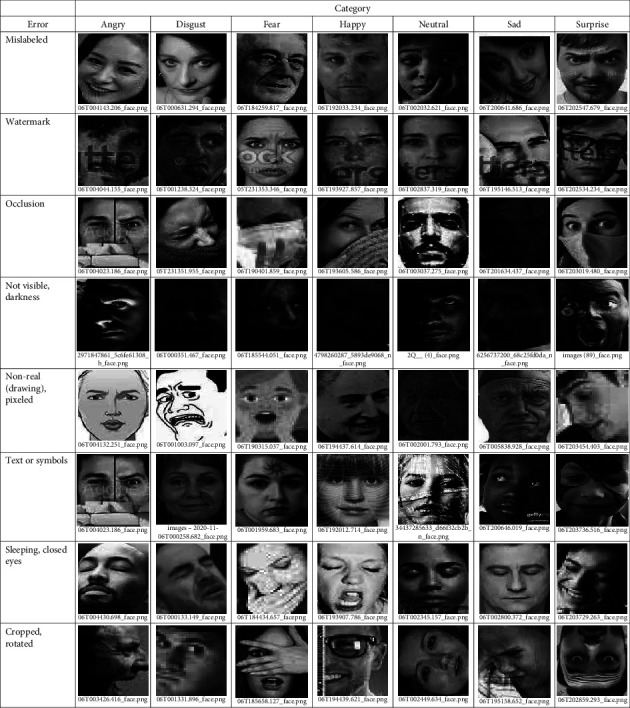
Some errors in the NHFI dataset.

**Figure 5:**
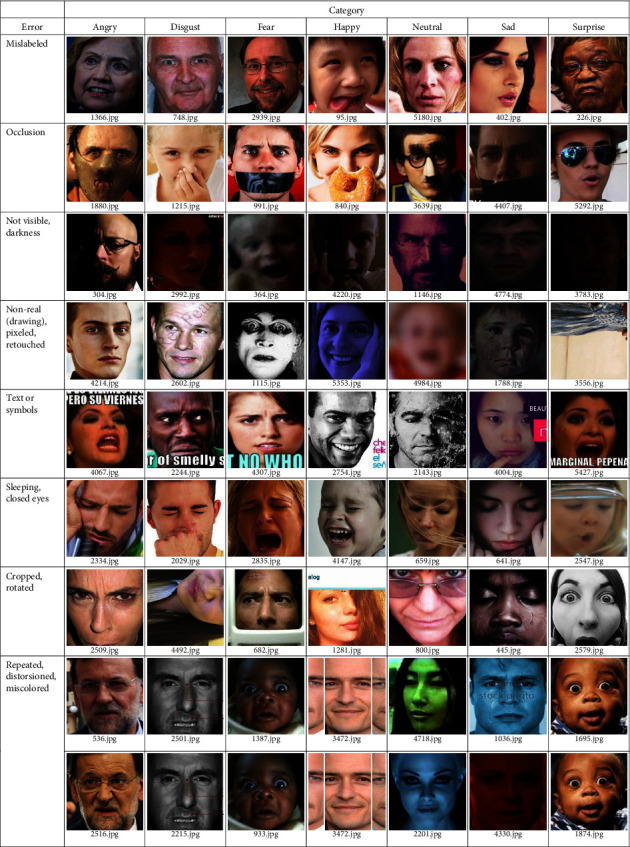
Some errors in the AffectNet dataset.

Search for facial images with errors follows the flowchart shown in Figure 2. We reused the CNN for facial expression recognition designed by Akshit Bhalla [27]. During the training on each dataset, we monitored the accuracy of the validation set at each iteration (epoch) to save the best model parameters. This model is used to perform the prediction on all the images of the validation set. The confusion matrix is obtained from these predictions, where the off-diagonal positions allow us to identify the failures and their corresponding images. As a result, we have a smaller set of images in each class that is stored in a separate folder. We then visually reviewed to select examples of mislabeling and irrelevant images with their respective file names (Figures 3–5).

In this section, we examined the problems of the FER datasets, which can be summarized as class imbalance, the existence of a significant number of images that are irrelevant, or that do not correspond to the correct category. Combined or separately, these problems cause the performance of a FER model to degrade considerably, as well as learning to be biased in favor of the dominant classes [8, 9, 18, 22]. Therefore, the search for more convenient architectures and configurations for recognition models is a waste of time when the data used are of low quality. Firstly, it is necessary to address these problems to improve the datasets. Dealing with both the imbalance and the irrelevant images involves changing the size of the original dataset. Our work focuses on the problem of misclassification by keeping the number of available images of the dataset. To this end, we propose a novel data-centric method based on deep learning for the automatic relabeling of facial images.

## 4. Proposed Method

Our goal is to achieve increased accuracy in facial expression recognition through deep learning by previously improving the dataset used. We proposed a data-centric approach that specifically addresses the misclassification typically encountered in FER datasets. This drawback is likely the most influential in the lower performance of recognition models in the wild scenarios. Since a visual inspection of every facial image in a dataset would be an extremely time-consuming and tedious task, we designed a method to automatically reclassify images of a dataset and improve the performance of a FER model.

### 4.1. Workflow

**Figure 6:**
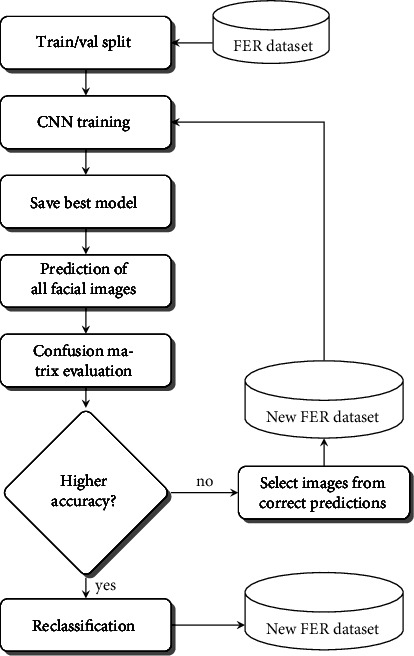
Workflow to automatically reclassify a FER dataset.

The proposed method consists of a series of steps represented by a workflow diagram in Figure 6.

1. The dataset is organized in a folder-based structure, where each facial expression category is a folder containing the corresponding facial image files.
2. Split the dataset into training and validation subsets, with the same folder and file structure. The training subset is larger and has the images to fit the model, whereas the validation subset is used to evaluate the model at training time. We omit a test subset because as many images as possible are needed for the next step. Thus, the input is ready for the deep learning model.
3. A CNN created from scratch or pretrained via the transfer learning technique is trained on the FER dataset. Both alternatives are shown in this work. Training is an iterative optimization process in which the model reduces an error as it learns to associate images and category labels.
4. The training is monitored to save in a model file the parameters (weights and biases) corresponding to the iteration (epoch) of the best validation accuracy.
5. The best model is used to perform the prediction of all facial images in the dataset. The results obtained allow us to generate the confusion matrix.
6. The confusion matrix is evaluated considering a good dataset when the precision of each category exceeds 90% or the numbers outside the main diagonal are single digits. Several successive trainings will be necessary to meet these criteria.
7. The correct predictions on the main diagonal of the confusion matrix allow us to select the corresponding facial images, which will form a smaller but much more reliable version of the dataset.
8. The new version of the dataset is automatically divided into training and validation subsets, and training is performed with the same CNN. The process is repeated until the conditions established for a good dataset are reached.
9. The last saved model performs the prediction of facial expression for all images in the original dataset. The result is the automatic reclassification generating a new distribution with all facial images.

In summary, we propose a process of iterative trainings to create successively more refined versions of the dataset. Each version is smaller, only the correct predictions of facial expression are included, but maintains a significant number of images. At the last training, a much more reliable dataset is obtained, as well as a model that produces a low number of incorrect predictions (single-digit values for each class). The convolutional network is fixed in terms of its architecture and hyperparameters along this process.

The key idea is that feature extraction is a crucial part of a FER system, and the expression classification accuracy will improve with an effective extraction of facial features [10, 11]. The progressive refinement of the dataset produces a smaller number of images in each training, but with less variability of the gestures of the faces. Therefore, the model can capture more distinctive features of each class gradually. As a consequence, it is possible to increase intraclass similarity and enlarge interclass differences within a dataset, thereby improving the accuracy of facial expression recognition in real-world scenarios.

### 4.2. Models

We leverage CNNs, current state-of-the-art tools in Computer Vision, for facial expression prediction in images. The design of CNNs imitates the human visual system, where a convolutional part would be the eyes of the network whereas a classifier part would be the brain, which decides the class of the object. CNNs can be created from scratch or pretrained using the transfer learning technique. In this work, we demonstrate the use of both alternatives, describing the architecture implemented for each of the datasets selected.

#### 4.2.1. FER2013

**Figure 7:**
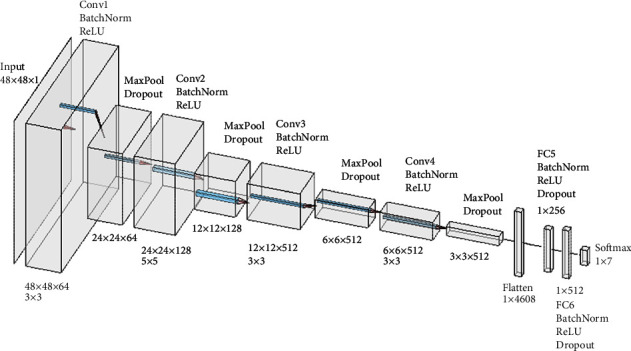
Architecture of the CNN for the FER2013 dataset.

We reutilized the CNN presented on the Kaggle site (https://www.kaggle.com/bhallaakshit/facial-expression-recognition), whose performance has shown good results in the task of facial expression recognition on this dataset (Figure 7).

The 48 × 48 pixel grayscale input image is passed through 4 convolutional layers, each layer applies a number of filters (kernels) to generate feature maps that include hierarchically detected patterns, from the simplest to the most complex. Here, 64, 128, 512, and 512 filters of size 3 × 3, 5 × 5, 3 × 3, and 3 × 3 pixels, respectively, are applied. A ReLU activation function then turns the negative values to zero and maintains the positive values. Next, a max-pooling operation reduces the image dimensions by half, but preserves the found features. Batch normalization stabilizes the result of a convolution whereas dropout enables the active participation of all neurons in the learning process. Both are recommended regularization techniques to avoid possible overfitting. The flatten operation converts the feature maps into a vector of values suitable as input for the classifier, which is a traditional fully connected neural network with an input layer that receives the features in vector shape, two hidden layers of 256 and 512 neurons, and an output layer with a Softmax activation function for 7 probability values, one for each facial expression class.

#### 4.2.2. NHFI

We tested the same CNN model with this dataset, however, the results after the first filtering indicated an insignificant increase in accuracy (approx. 1.5%) as shown in Table 3.

**Figure 8:**
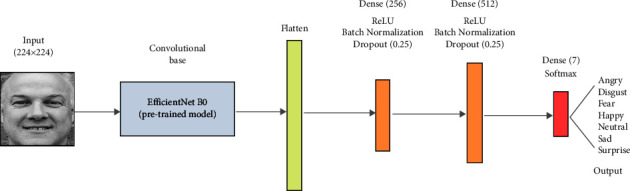
Architecture of the CNN with transfer learning for the NHFI dataset.

Therefore, we searched for other architectures to achieve higher accuracy. A model using the transfer learning technique showed the best performance for this dataset. In the first filtering, the accuracy improved from 0.5597 to 0.8367 (27.7%) as opposed to 1.5% with the CNN from scratch. Thus, we were able to demonstrate that the proposed method works for both cases (pretrained and from scratch models). With transfer learning, the training phase will be much faster, since we only train the classifier parameters while keeping fixed the convolutional base that would have already learned features that are useful for most computer vision problems. The structure is presented in Figure 8.

The model is based on the *EfficientNet*, a very popular CNN pretrained on the ImageNet dataset [28]. We used version *B0*, whose convolutional base is kept for feature extraction. The advantage is that the image with the original size of 224 × 224 pixels is accepted as input. The classifier receives the features in the form of a flattened vector to decide the class to which the input image belongs by means of a fully connected neural network with two dense layers of 256 and 512 neurons, to which the ReLU activation function is applied, plus the batch normalization and dropout regularization techniques to reduce possible overfitting. The Softmax function in the last dense layer outputs a distribution of probabilities corresponding to each of the 7 categories of facial expression.

#### 4.2.3. AffectNet

We performed several tries with different architectures to determine the most suitable CNN for this dataset. The best result was obtained with the CNN used for the FER2013 dataset (Figure 7). It is only necessary to change the size and color mode of the AffectNet images from 224 × 224 pixels in RGB to 48 × 48 pixels in grayscale. This conversion is performed automatically using the image generator of Python.

## 5. Experiments

The core of the experimentation is the run of trainings of each CNN-based model on the respective dataset. The main characteristics of the computational platform used are a processor Intel(R) Core(TM) i9-7920X, 2.90 GHz, RAM 64 GB, GPU NVIDIA GeForce RTX208 with RAM 12 GB, and the operating system Linux Ubuntu 18.04.5 LTS. The CNN architectures described in the previous section are implemented using Python version 2.7.17, supported by standard libraries such as OS, NumPy, and Matplotlib, to manage directories and files, numeric arrays, and visualization, respectively. For deep learning work, we used libraries such as TensorFlow, Keras, and scikit-learn, as well as the Image Data Generator utility for image preprocessing.

The learning process is aimed at model learning to associate facial images and labels of expression categories. A series of values known as hyperparameters must be explicitly defined by the programmer before training. There are no fixed rules for determining these values, they are the result of several tests to find the most convenient ones.  Table 4 shows the hyperparameters for each model and dataset, which are maintained for all experiments.

Our method of dataset refinement required five successive trainings for each dataset to meet the quality criteria. At each training, the model is fed with the facial images from the training subset of each dataset in batches of 64 images (batch size). We used the Image Data Generator utility from Keras to work with an image generator in batches, due to the large number and size of the images would cause a storage problem in memory. It also allows us to pass the images directly to the training model from directories, as well as automatically labeling the image with the respective category, and performing data augmentation. For each batch, predicted and actual labels are compared, obtaining a *loss* and an *accuracy* using the *categorical_cross entropy* function. Backpropagation and *Adam* (based on gradient descent) algorithms are applied to update the model weights according to the *learning rate* value. When all batches are completed, one *epoch* is accomplished, i.e., one iteration of all training images. The accuracy and loss values are measured after each epoch using the images from validation the subset. One hundred epochs have been run for FER2013, whereas for NHFI and AffectNet fifty epochs were sufficient to know the maximum level of accuracy since beyond this value, the behavior of the model remains practically stable and an improvement is not appreciable. The callback utility from Keras is leveraged to perform certain actions during training such as setting a checkpoint and reducing the learning rate. The model will only be saved to disk if the validation accuracy in the current epoch is greater than what it was in the last epoch. On the other hand, the learning rate tells us how much the weights will be updated each time, and is often between 0 and 1. It will decrease from an initial value to a minimum if the loss does not improve after a certain number of epochs, which usually results in better training.

## 6. Results

The results of the experimentation are presented graphically by means of learning curves and confusion matrices, whereas the numerical metric used for comparison is the validation accuracy. These tools allow us to evaluate the performance of the model and the improvement of the dataset. During the training and validation of each model, loss and accuracy values have been collected, respectively. This generates the so-called* learning curves*, where the horizontal axis represents the number of epochs and the vertical axis represents either the accuracy or the error. The confusion matrix, also known as the error matrix, is a table to visualize the model performance as presents information about actual and predicted classifications carried out by a classifier model. Rows represent the instances of actual classes, whereas columns represent the instances the classifier predicts [29]. From this matrix, several performance metrics can be obtained, however, we focus on *accuracy*, which compares the number of correct predictions (on diagonal) divided by the total number. The results obtained for each one of the analyzed datasets are presented next.

### 6.1. FER2013

**Figure 9:**
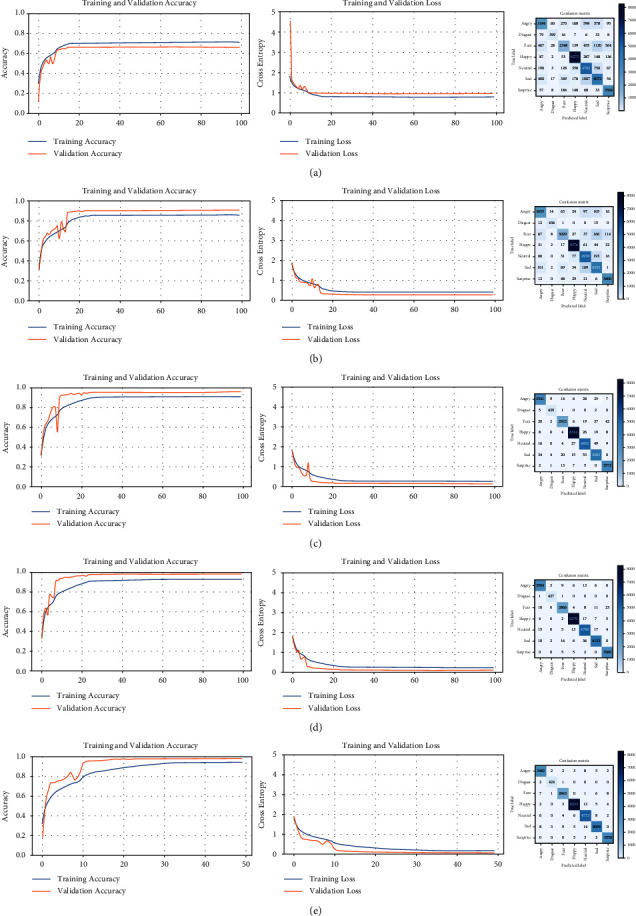
Learning curves and confusion matrices for five successive trainings of the FER2013 dataset. (a) Training #1, (b) training #2, (c) training #3, (d) training #4, and (e) training #5.

The learning curves and confusion matrix for each of the five trainings required for the FER2013 dataset are shown in Figure 9. For each training (including validation), the following are presented: the accuracy curves (left), the loss curves (middle), and the corresponding confusion matrix (right). As more trainings are performed, the accuracy curves (training and validation) reach higher values, whereas the loss curves are decreasing in height and near to zero. In addition, the pairs of curves are very close to each other in all the graphs. Therefore, the accuracy of the model is higher, the error is lower, and there is no overfitting. This ideal behavior is the product of successive filtering of the dataset. The confusion matrices include the predictions of facial expressions for all the images in the dataset used for each training. The progressive trainings cause the desired effect in each matrix, that is, to reduce the values outside the main diagonal and to increase the values in this diagonal. The model is each time more accurate because wrong predictions are discarded in subsequent training. As a result, more distinctive features of each class are captured. In this way, the intraclass variability of the facial images is decreased and the interclass variability is increased.

The process of the dataset refinement is summarized in Table 5. Five trainings (four filtering operations) on the FER2013 dataset were necessary to achieve the expected performance metric (validation accuracy). Another training was not necessary because there is no significant improvement in the accuracy. The number of images gradually decreases, but it is still considerable for each training. The model with the highest accuracy (97.7%) has captured the most distinctive features of each facial expression category and is convenient for reclassifying all images in the dataset. The *predict()* method is used to assign the category of every facial image of the original dataset, generating a new distribution of the FER2013 dataset. The comparison is presented in Table 6.

**Figure 10:**
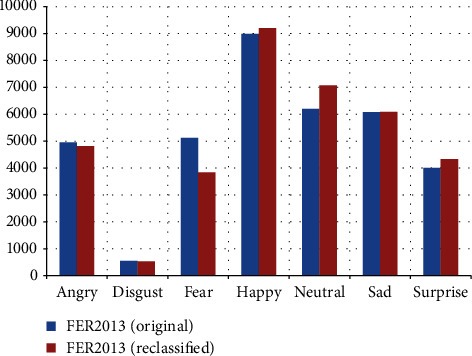
Graphical comparison of both distributions.

Figure 10 shows that the categories “disgust” and “sad” have minimal variation, those of “angry,” “happy,” and “surprise” vary moderately, and the most affected categories are “fear” (decreasing) and “neutral” (increasing), indicating that the original FER2013 dataset suffers from misclassified facial images, especially between these two categories.

**Figure 11:**
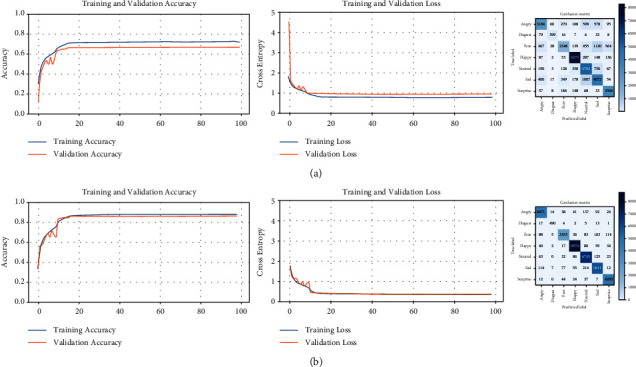
Comparison between the original and reclassified FER2013 dataset. (a) FER2013 dataset (original) and (b) FER2013 dataset (reclassified).

The decisive test of the effectiveness of our method is to train the same CNN on the reclassified FER2013 dataset.  Figure 11 shows that better learning curves are obtained, as well as the confusion matrix indicates more correct and fewer incorrect predictions. Higher accuracy and lower loss are verified in Table 7.

The results confirm a more reliable dataset keeping the number of images. The reclassified FER2013 enabled a very significant increase in the validation accuracy of the model by 20.45% and the loss is much lower (0.34). The training accuracy is acceptable (88.76%), very close to the validation accuracy and the loss is lower. There is no overfitting and no significant difference between training loss and validation loss. All the categories show improved accuracy, in particular, there is a remarkable improvement for “angry” (an increase of 26%), “fear” (an increase of 38%), and “sad” (an increase of 25%), i.e., those that showed the most overlapping or confusion. According to the experiments, only 40 epochs in each training would be sufficient, since the behavior remains practically stable beyond this number.

### 6.2. NHFI

**Figure 12:**
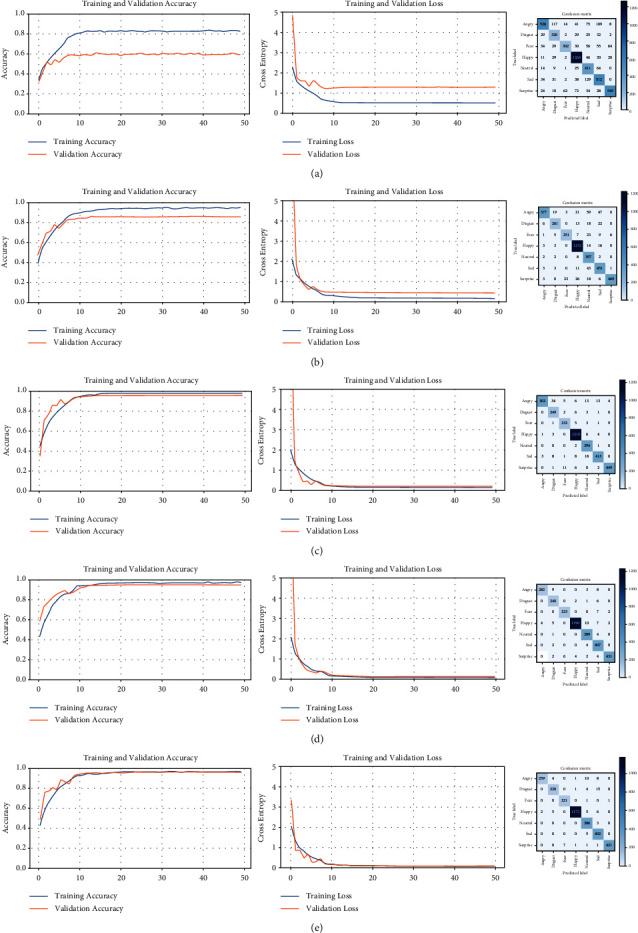
Learning curves and confusion matrices for five successive trainings of the NHFI dataset. (a) Training #1, (b) training #2, (c) training #3, (d) training #4, and (e) training #5.

The accuracy curves for the NHFI dataset (left side in Figure 12) start quite separated from the other, evidencing the presence of overfitting, but as the trainings are performed, the curves become closer and reach high accuracy, similar to the loss curves, but in the opposite direction, becoming closer and nearer to the horizontal axis. The confusion matrices show higher values on the main diagonal and lower values of this diagonal, indicating the progressive improvement of the model accuracy, as well as the quality of the dataset used in each training. Despite successive discarding of incorrect predictions, the number of images is significant with respect to the original quantity.  Table 8 shows the evolution of the trainings on the NHFI dataset.

**Figure 13:**
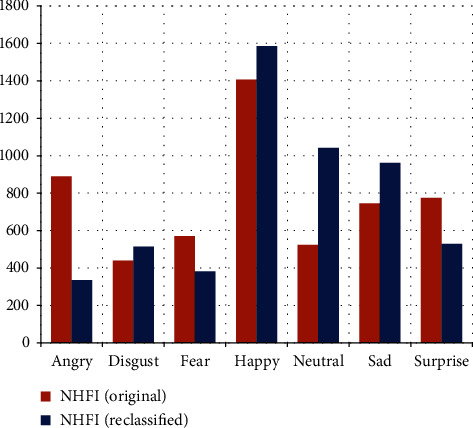
Graphical comparison of both distributions.

The reclassification of the original NHFI dataset is performed with the highest accuracy model (96.66%). A new distribution of the dataset is generated, which is shown in Table 9. In Figure 13, we can note that the “angry” and “neutral” categories had the greatest changes, indicating that these categories have the most intraclass variability in the original dataset.

**Figure 14:**
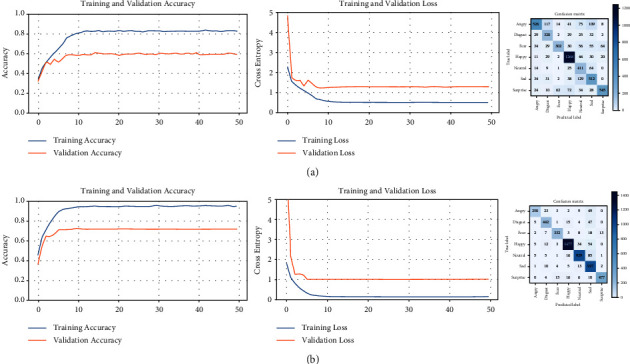
Comparison between the original and reclassified NHFI dataset. (a) NHFI dataset (original) and (b) NHFI dataset (reclassified).

To demonstrate improved recognition, the same CNN is trained on the reclassified NHFI dataset and the result is compared to the original dataset (Figure 14). The overfitting was not reduced, but the accuracy is higher, both in the training and validation subsets. The loss is decreased for the reclassified NHFI dataset, as well as the values off-diagonal from the confusion matrix.

The performance results for the original and reclassified distributions of the NHFI dataset are presented in Table 10. We have been able to significantly increase the accuracy in both training and validation subsets, by 18.74 and 14.47%, respectively. Except for the “angry” and “happy” categories, the validation accuracy is highly increased in the rest of the categories, particularly in the “sad” category from 49 to 85%. The methodology based on successive filtering with a transfer learning model has been successfully applied on a different dataset than FER2013.

### 6.3. AffectNet

The version of the AffectNet dataset we selected contains 287401 images with a large imbalance between the categories (Figure 1(c)). Training on this dataset can lead to biases and erroneous assessment of model accuracy. Therefore, we applied downsampling to balance all categories by considering the one with the lowest number of images. The “disgust” category limited the other categories with 4300 images, of which 3800 have been randomly selected for training by using the split-folders (https://pypi.org/project/split-folders/) library, whereas 500 images by default come with the dataset for validation. The balanced version is shown in Table 11.

**Figure 15:**
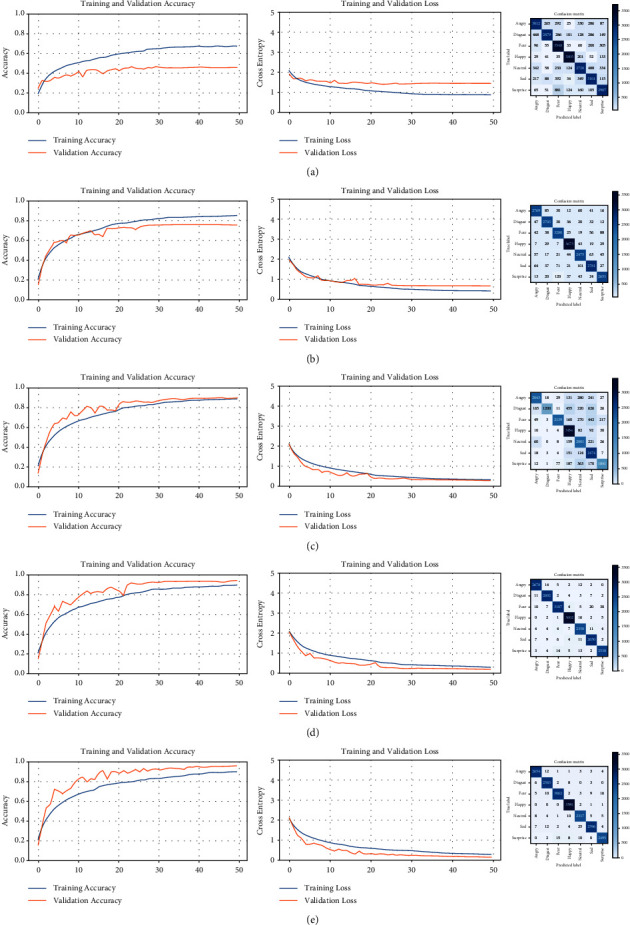
Learning curves and confusion matrices for five successive trainings of the AffectNet dataset. (a) Training #1, (b) training #2, (c) training #3, (d) training #4, and (e) training #5.

The refinement process is performed on this balanced version of the AffectNet dataset. The accuracy curves for the training and validation subsets (Figure 15) start with a small separation, which decreases as successive trainings are performed, even the validation curve finishes outperforming the training curve in accuracy. The same behavior, but in the opposite direction, is presented for the loss curves. The values on the main diagonal of the confusion matrix increase with each training and decrease off this diagonal, indicating a higher accuracy of the model due to a better dataset. The evolution of the successive training is summarized in Table 12.

The model in the last training reaches a higher validation accuracy (95.9%), which allows us to reclassify the balanced dataset. A new distribution of the AffectNet of 30100 images is generated, whose number of images per category is presented in Table 13.

**Figure 16:**
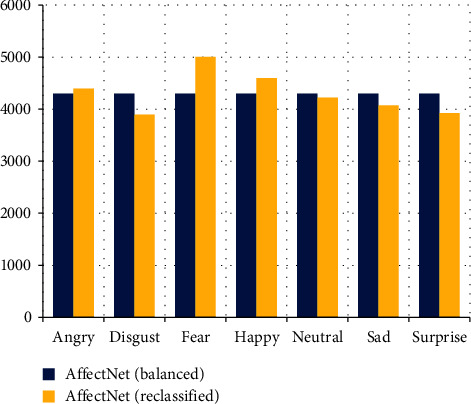
Graphical comparison of both distributions.

After the reclassification of the balanced dataset, the new distribution is imbalanced (Figure 16). The categories of happy and fear have increased significantly, whereas the category of anger has increased slightly. In the remaining cases, there is a decrease, mainly in the categories of disgust and surprise.

**Figure 17:**
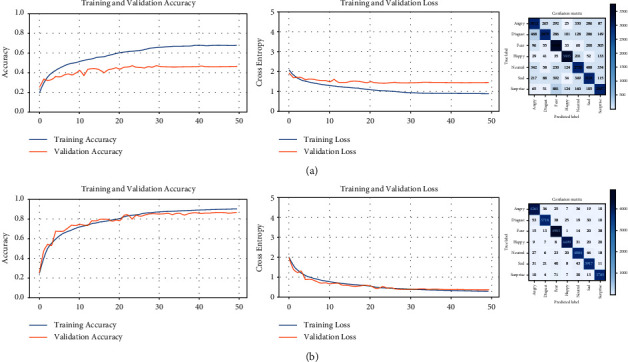
Comparison between the balanced and reclassified AffectNet dataset. (a) AffectNet dataset (balanced), (b) AffectNet dataset (reclassified).

Next, the CNN-based model is trained on the new version of the AffectNet dataset to verify that our method works.  Figure 17 presents the learning curves of both versions of the dataset, where the new AffectNet (Figure 17(b)) allows to achieve better performance with higher accuracy.

There is a notable improvement in accuracy compared to the first training of the balanced dataset (Table 14). Due to downsampling, the split ratio is 88 and 12%, for the training and validation subsets, respectively. For the new version of the dataset, the proportion is 80 and 20% and having more validation images, the accuracy percentage is almost duplicated (39.66%).

We successfully applied our method to a smaller and more balanced version of the AffectNet dataset. The purpose is to improve the original AffectNet dataset, which is larger and imbalanced. To this end, the last trained model is used to reclassify the facial images in the full version of AffectNet. The new distribution is presented in Table 15.

**Figure 18:**
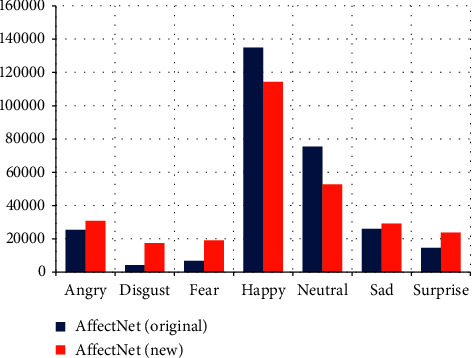
Graphical comparison of both distributions.

The bar plot in Figure 18 shows that the shape of the distribution of the new reclassified AffectNet is similar, however, there is a clear increase of images in the categories of fear, disgust, and surprise. This suggests that many facial images of these categories were misclassified as happy or neutral.

**Figure 19:**
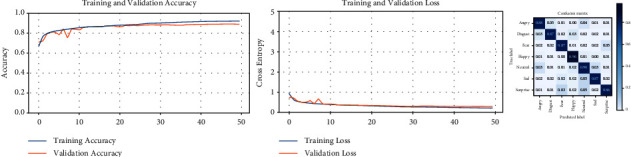
The learning curves and confusion matrix for the reclassified AffectNet dataset.

The following demonstrates the improved performance in facial expression recognition. The reclassified version of the AffectNet dataset is used to train the same CNN-based model, resulting in the learning curves and confusion matrix displayed in Figure 19. The accuracy curves of the training and validation subsets are increasing from the first epoch and reach a very high level, close to 90%. Also, both curves stay very near to each other. The error curves decrease together to levels near to zero, which is desirable. By using the validation results as suggested by the creators of the dataset, we calculated the accuracy with the *evaluate()* method and generated the normalized confusion matrix. The accuracy on the reclassified validation set is 89.17%, and for each facial expression, category fluctuates between 86% and 96%, which demonstrates a high rate of recognition and no bias for any of the categories as opposed to the original dataset. This behavior confirms better FER performance on the reclassified AffectNet dataset.

Finally, in Table 16, the results of the proposed method are compared with the state-of-the-art performance on the same datasets used in the present work. These are single network models that did not use extra images to the existing ones in the datasets. In all cases, our reclassified versions of the datasets allow us the highest accuracy values for both the model from scratch and transfer learning. For the novel NHFI dataset, there is no formal report on classification accuracy, so we set as a baseline the accuracy achieved by the transfer learning model on the original dataset and contrast it with the reclassified version. These results demonstrate the effectiveness of our data-centric method, as it improves the performance of the FER models even achieving state-of-the-art accuracy values.

## 7. Conclusions and Future Work

Facial expression recognition in the wild is a challenging problem for computer systems. Promising results have been achieved with deep learning methods, where the model and the data share responsibility. The vast majority of the research is oriented towards designing better models, which is not sufficient when the data suffers from drawbacks. One of the most influential problems in FER datasets is misclassification. In this work, we presented and implemented a method to reclassify all the facial images of a dataset by generating a new distribution that increases the accuracy of the FER models. The proposed method keeps the convolutional network fixed and iteratively improves the data over successive trainings. After each training, the dataset is evaluated with the confusion matrix, and the facial images corresponding to the correct predictions (on-diagonal) are selected to form the subsequent training data. This process gradually generates a more accurate model and more distinctive features for each category of facial expression. The model from the last training is used to reclassify all the images creating a new distribution of the dataset. We experimented with popular FER datasets and CNNs created from scratch and Transfer Learning. The increase in validation accuracy by 20.45%, 14.47%, and 39.66%, for FER2013, NHFI, and AffectNet, respectively, corroborates the efficacy of the proposed method. The results suggest that the quality and size of the dataset determine the most appropriate type of model. NHFI is a small and better-annotated dataset, so a pretrained model is convenient, unlike larger and lower quality datasets, which need a model from scratch, with longer training and more parameters. The reclassified versions of these datasets maintain the same number of images as the original dataset, but with less overlapping between categories, and less variability within the same category of facial expression. This allows us to achieve the state-of-the-art performance of single network FER models with 86.71%, 70.44%, and 89.17%, for FER2013, NHFI, and AffectNet, respectively. The recognition rates improved most significantly for the largest and lowest classified datasets, i.e., the proposed method works best for datasets with a high level of misclassified images. The refinement process of the dataset would enable several models to work well, not only diverse architectures of CNN, but others such as the transformer. Our proposal, beyond the application to the FER domain, is also useful for a variety of computer vision problems when the data are images. Furthermore, it can serve as a debugging tool in the automatic collection of image datasets. We maintained the size of the dataset, considering that quantity is important. However, there are irrelevant images that should be removed and the imbalance could be addressed with data augmentation or GANs. We believe that these contributions would improve the quality of the dataset and the accuracy of the models. Therefore, a methodology for automatic learning should consider the quality of the dataset as a prerequisite to the search for better network architectures and model configurations.

## Figures and Tables

**Table 1:** Datasets considered and their main characteristics.

Characteristic	FER2013	NHFI	AFFECTNET
Number of images	35886	5558	287401
Expression model	Discrete	Discrete	Discrete/continuous
Categories	7	8	8
Type	2D facial image	2D facial image	2D facial image
Labelers	Humans	Humans	Automated and humans
Balanced	No	No	No
Resolution (pixels)	48 × 48	224 × 224	224 × 224
Color	Grayscale	Grayscale	RGB color
Format	JPG	PNG	JPG
Space	300 MB	50 MB	4 GB
Availability	Free	Free	Under request
Data source	Internet	Internet	Internet
Size	Mid	Small	Large
Environment	In-the-wild	In-the-wild	In-the-wild
Year	2013	2020	2017
Structure	CSV file	Folders and files	Image and NumPy files
Subsets (%)	Train/test (80/20)	None	Train/val (99/1)

**Table 2:** Distribution of categories and number of images in FER datasets.

Dataset	Angry	Disgust	Fear	Happy	Neutral	Sad	Surprise	Total
FER2013	4953	547	5121	8988	6198	6077	4002	35886
NHFI	890	439	570	1406	524	746	775	5350
AffectNet	25382	4303	6878	134915	75374	25959	14590	287401

**Table 3:** Refinement of the NHFI dataset using the CNN from scratch.

Training	Images (train)	Images (val)	Total	Accuracy
1	4278	1072	5350	0.5732
2	3211	616	3827	0.5885

**Table 4:** Training hyperparameters set for our experiments.

Hyperparameter	FER2013	NHFI	AffectNet
Input shape	48, 48, 1	224, 224, 3	48, 48, 3
Train-val (%)	80–20	80–20	80–20
Batch size	64	64	64
Learning rate	0.01 to 0.00001	0.01 to 0.00001	0.001 to 0.00001
Optimizer	Adam	Adam	Adam
Loss function	categorical_cross entropy	categorical_cross entropy	categorical_cross entropy
Metrics	Loss and accuracy	Loss and accuracy	Loss and accuracy
Number of classes	7	7	7
Epochs	100	50	50
Data augmentation	Yes	No	No
Number of training	5	5	5

**Table 5:** Summary of experimental results for the FER2013 dataset.

Training	Images (train)	Images (val)	Total	Accuracy
1	28708	7178	35886	0.6702
2	25415	4810	30225	0.9089
3	24001	4379	28380	0.9582
4	23654	4179	27833	0.9761
5	23488	4079	27567	0.9770

**Table 6:** Distribution of the original and reclassified FER2013 dataset.

Dataset	Angry	Disgust	Fear	Happy	Neutral	Sad	Surprise	Total
FER2013 (original)	4953	547	5121	8988	6198	6077	4002	35886
FER2013 (reclassified)	4817	532	3842	9202	7074	6090	4329	35886

**Table 7:** Comparison of the training results for the original and reclassified FER2013 datasets.

Dataset	Images (training)	Images (validation)	Total	Accuracy
FER2013 (original)	28708	7178	35886	0.6626
FER2013 (reclassified)	28708	7178	35886	0.8671

**Table 8:** Summary of experimental results for the NHFI dataset.

Training	Images (train)	Images (val)	Total	Accuracy
1	4278	1072	5350	0.5597
2	3284	600	3884	0.8367
3	2936	502	3438	0.9382
4	2786	471	3257	0.9533
5	2713	449	3162	0.9666

**Table 9:** Distribution of the original and reclassified NHFI dataset.

Dataset	Angry	Disgust	Fear	Happy	Neutral	Sad	Surprise	Total
NHFI (original)	890	439	570	1406	524	746	775	5350
NHFI (reclassified)	336	514	383	1585	1042	962	528	5350

**Table 10:** Comparison between the original and reclassified NHFI dataset.

Dataset	Images (train)	Images (val)	Total	Train_acc	Val_acc
NHFI (original)	4278	1072	5350	0.7676	0.5597
NHFI (reclassified)	4278	1072	5350	0.9550	0.7044

**Table 11:** Balanced distribution of the AffectNet dataset.

Subset	Angry	Disgust	Fear	Happy	Neutral	Sad	Surprise	Total
Train set	3800	3800	3800	3800	3800	3800	3800	26600
Val set	500	500	500	500	500	500	500	3500
Total	4300	4300	4300	4300	4300	4300	4300	30100

**Table 12:** Summary of results for the balanced AffectNet dataset.

Training	Images (train)	Images (val)	Total	Accuracy
1	26600	3500	30100	0.4686
2	17587	4393	21980	0.7612
3	16268	4064	20332	0.9016
4	15843	3956	19799	0.9401
5	15617	3902	19519	0.9590

**Table 13:** Distribution of the balanced and reclassified AffectNet dataset.

Dataset	Angry	Disgust	Fear	Happy	Neutral	Sad	Surprise	Total
AffectNet (balanced)	4300	4300	4300	4300	4300	4300	4300	30100
AffectNet (reclassified)	4394	3893	5004	4594	4224	4071	3920	30100

**Table 14:** Comparison of the training results for the balanced and reclassified AffectNet datasets.

Dataset	Images (train)	Images (val)	Total	Train_acc	Val_acc
AffectNet (balanced)	26600	3500	30100	0.6763	0.4686
AffectNet (reclassified)	24084	6016	30100	0.9013	0.8652

**Table 15:** Distribution of the original and new AffectNet datasets.

Dataset	Angry	Disgust	Fear	Happy	Neutral	Sad	Surprise	Total
AffectNet (original)	25382	4303	6878	134915	75374	25959	14590	287401
AffectNet (new)	30827	17475	19145	114275	52760	29160	23759	287401

**Table 16:** Comparison of state-of-the-art performance on the FER datasets considered.

Dataset	Work	Model	Accuracy (%)
FER2013	[9]	VGG fine tuning	73.28
FER2013 (reclassified)	Ours	CNN from scratch	86.71
NHFI	Ours	EfficientNet-B0 transfer learning	55.97
NHFI (reclassified)	Ours	EfficientNet-B0 transfer learning	70.44
AffectNet	[30]	CNN-attention mechanism	65.69
AffectNet (reclassified)	Ours	CNN from scratch	89.17

## Data Availability

The code developed from this study is available in the GitHub repository (https://github.com/cimejia/FER-datasets/), whereas the datasets generated (except for the reclassified version of AffectNet) are available from the corresponding author upon request.
